# The Problem of Constipation Solved

**Published:** 1889-03

**Authors:** 


### THE PROBLEM OF CONSTIPATION SOLVED.

We believe the immortal Marion Sims was the first to point out the hydragogue properties of glycerine locally applied. He introduced a tampon of cotton, saturated with glycerine, and applied it to an engorged *os uteri*. On removing it the next day he was surprised to find a gush of water, and the phlogosis of the parts entirely relieved. The lady, also, called his attention to the fact that a copious discharge of water had occurred during the whole time after the application had been made. Since which time, glycerine has been used for similar purposes, and with satisfactory results. What suggested its use as enemata for constipation we do not know; but the idea, based on the above now known property, is certainly rational, and it would seem that it could be carried further, and made to extend to the treatment of acute dysentery, for, on a similar principle we give epsom salts, its well known property of abstracting water from the engorged blood vessels of the rectum.

But the use of glycerine in its liquid state for enemata was unsatisfactory and inconvenient. Messrs. Park, Davis & Co. have, with their usual sagacity, taken advantage of the idea, and have given to the profession a glycerine suppository. It is a beautiful preparation, and accomplishes the desired purpose most admirably. The suppository is in the shape of a double cone, and may be cut into two or more pieces, as it is not the quantity introduced, merely, that does the work; we find that even a small portion, the tip end of one will produce a free fecal action in a very few moments,. Moreover, the introduction into the bowel is not at all painful, nor is there produced any straining or tenesmus; the action is free, satisfactory and natural. It appears to do more than empty the rectum; a peristalsis of the upper bowel is induced, perhaps by reflex action. We have given the suppository a trial, and find that its effect is almost immediate. In cases of fecal impaction in the lower bowel, it relieves at once, by softening the mass. In hemorrhoids, it would seem to offer brilliant attractions; and in dysentery a little cocaine might be added.

This is not a puff for Park, Davis & Company by any means; though they deserve the thanks of the profession, but is written for the purpose of recording what we consider one of the greatest advances in therapeutics made in many years. The suppositories must be kept in a dry place, as the great affinity of glycerine for water causes them to absorb moisture, by which they are decomposed.

